# Sex Differences in Medicine Acceptability: A New Factor to Be Considered in Medicine Formulation

**DOI:** 10.3390/pharmaceutics11080368

**Published:** 2019-08-01

**Authors:** Fabrice Ruiz, Alexander Keeley, Patrick Léglise, Catherine Tuleu, Célia Lachuer, Jean-Paul Rwabihama, Nathalie Bachalat, Imad Boulaich, Fattima Abdallah, Maité Rabus, Annie-Claude Ribemont, Hugues Michelon, Amélie Dufaÿ Wojcicki, Mine Orlu, Thibault Vallet, Vincent Boudy

**Affiliations:** 1 ClinSearch, 110 Avenue Pierre Brossolette, 92240 Malakoff, France; 2 Department of Pharmaceutics, UCL School of Pharmacy, 29-39 Brunswick Square, London WC1N 1AX, UK; 3 Hôpital Joffre Dupuytren, Hôpitaux Universitaires Henri Mondor, Assistance Publique-Hôpitaux de Paris (AP-HP), 1 rue Eugène Delacroix, 91210 Draveil, France; 4 Hôpital Sainte-Périne, Hôpitaux Universitaires Paris Ile-de-France Ouest, Assistance Publique-Hôpitaux de Paris (AP-HP), 11 rue Chardon-Lagache, 75016 Paris, France; 5 Unité de R&D Galénique, Agence Générale des Equipements et Produits de Santé (AGEPS), Assistance Publique-Hôpitaux de Paris (AP-HP), 7 rue du Fer À Moulin, 75005 Paris, France

**Keywords:** medicine, acceptability, palatability, taste assessment, older population, formulation, Alzheimer, sex, CAST—clinsearch acceptability score test, brief-access taste aversion

## Abstract

Palatability is a recognized driver of medicine acceptability in pediatrics but deemed less relevant in older populations due to sensory decline. Preliminary findings from an observational study implicated palatability problems with one Alzheimer’s medicine. Among 1517 observer reports combining multiple measures on medicines uses in patients aged over 64, we focused on two original formulations of memantine (Ebixa^®^, tablets (*n* = 25) and oral solution (*n* = 60)). Evaluations were scored with an acceptability reference framework (CAST), the rodent Brief Access Taste Aversion (BATA) model tested aversiveness. Focusing on women treated with Ebixa^®^ (*n* = 54), the oral formulation sub-group was classified as “negatively accepted”, while the coated tablet was associated with the “positively accepted” cluster. In men, both formulations belonged to the “positively accepted” profile. Using BATA, the original oral solution was categorized as highly aversive/untolerated while solutions of excipients only were well tolerated. Furthermore, the number of licks was significantly lower in female than in male rats. These results revealed that medicine palatability remains important for acceptability in older populations. Moreover, converging results from humans and animal models highlighted that palatability profiles can significantly vary between the sexes. These drivers should be closely considered during drug development to enhance acceptability in this population.

## 1. Introduction

Adherence is crucial for obtaining optimum clinical benefits from any treatment. In the past decade, the World Health Organization (WHO) has devoted a full report to this concept [1] in which adherence to long-term therapy was reported to average only 50% in developed countries. The WHO report emphasized that this resulted in poorer outcomes while increasing health care costs. According to a systematic review [2], in the United States lack of adherence was estimated to have led to approximately 125,000 deaths and at least 10% of all hospitalizations annually. The authors also stated that the cost to the US healthcare system of non-adherence has been estimated between $100 billion and $289 billion per year. In 2018, the Organisation for Economic Co-operation and Development (OECD) addressed this subject in a working paper entitled “Investing in medication adherence improves outcomes and health system efficiency”. Based on data from across Europe in 2010, the OECD estimated that poor adherence to treatment led to nearly 200,000 premature deaths, and in terms of avoidable hospitalizations alone was responsible for 125 billion euros of government spending annually [3].

In this context, the European Medicine Agency (EMA) came to consider that patient acceptability is likely to have a significant impact on patient adherence and consequently on the safety and efficacy of medicinal products. Acceptability has been defined by the EMA as “The overall ability and willingness of the patient to use and its care giver to administer the medicine as intended”. To encourage developers to consider medicine acceptability for pediatric patients, the EMA dedicated a chapter to this multi-faceted concept in a recent guideline [4]. Key drivers of acceptability from product and patient characteristics were identified, palatability (and swallowability) was (were) first on the EMA’s list of product characteristics. Indeed, as many drugs have a bitter and often aversive taste, this poses a challenge to the development of palatable oral dosage forms [5]. However, when the EMA drafted a comparable reflection paper for the older population 3 years later, palatability was not addressed.

The epidemiology of diseases in the older population could support this omission of palatability from the list of important product characteristics for acceptability in the older population: the prevalence of dysgeusia increases with age [6,7,8]. However, as dysphagia is also a common disability in the older population, the use of oral drug delivery may be compromised with age [9]. In practice, liquid formulations have been developed for the older population to improve swallowability, but palatability has generally not been taken into consideration.

An Alzheimer’s disease treatment, memantine hydrochloride, exemplified this formulation strategy for older adults. The original medicine (Ebixa^®^, memantine hydrochloride) was formulated as both a coated tablet (10 and 20 mg) and an oral solution (10 mg/mL). Surprisingly, the oral solution was formulated without any flavoring agent [3].

Herein we have presented data contradicting these assumptions. Preliminary findings from an observational study on medicines acceptability in the older population demonstrated that the palatability of oral liquid pharmaceutical products remains a crucial acceptability driver, especially in older women (unpublished observations).

To confirm these results and limit the bias induced by variation of product characteristics we decided to focus our investigations on the two formulations of memantine hydrochloride cited above. The data collected during an acceptability study in older adults has been combined with results from an aversiveness study in animals to better understand the possible effects of sex and excipients.

## 2. Materials and Methods

### 2.1. Acceptability Study in Older Adults

#### 2.1.1. Data Source

The data presented here were collected in a multicenter, prospective, cross-sectional and strictly observational study conducted in France between October 2016 and May 2019. Patients were recruited at random on a voluntary basis at the recruiting centers. Approvals (CCTIRS n°16.390bis 12-SEP-2016) were obtained from the French advisory committee for data processing in health research and the data protection authority. Data collection was carried out in collaboration with a network of physicians and pharmacists in eight hospitals and eight nursing homes. The healthcare professional observing the first medicine use in older patients (65 years and over) following study inclusion, with the exception of infusions in which a catheter was already present, filled in a standardized web-questionnaire consisting of variables that explain and describe acceptability [10].

Healthcare professionals or clinical research associate reported information on the patient (e.g., sex, age, comorbidities), the treatment (e.g., the pharmaceutical product taken, the required dose), and the context of use (e.g., the person(s) in charge of preparing and administrating the medicine, the place and the time of medicine use) to investigate their influence on acceptability. Such information was generally taken from the patient’s medical record. In addition, observers were required to report multiple behaviors and events observed during the use of the medicine: the result of intake (the required dose fully, partly or not taken at all), the patient reaction during the administration (positive, neutral or negative reaction), the time needed to prepare (from opening any packaging to having a required dose of medication ready to use, including all handling and modifications), and the time needed to administer the required dose of medication (from a required dose of medication ready to use to the end of its intake). Preparation and administration time were classified as short (less than 20 s), medium (from 21 s to 1 min) or long (longer than 1 min). Furthermore, recourse to any methods to ease/achieve administration was reported: dividing the intake of a dose which cannot be taken as a whole, altering the intended use (manipulating dosage forms such as crushing tablets or opening capsules; use of a device not provided; use of another route/mode of administration), use of food/drink to mask a taste or ease swallowing, and use of restraint.

#### 2.1.2. Data Analysis

To study medicine acceptability we used CAST—ClinSearch Acceptability Score Test^®^ (CAST), a data driven approach [11] with a dedicated tool for the older population [10].

To build the acceptability reference framework, all the observed measures were included in the multivariate analysis without any weighting. A multiple correspondence analysis provided a three-dimensional acceptability map summarizing the key relationships between the observed measures. Subsequently, hierarchical clustering on principal components and k-means consolidation gathered the most similar evaluations into two clusters, defining coherent acceptability profiles: the “Positively accepted” and the “Negatively accepted” profiles. These profiles were materialized by a green and a red area on the acceptability map, respectively.

Herein we used the reference framework to score the acceptability of the drug products of interest: the original formulations of memantine hydrochloride (Ebixa^®^). The barycenter of the evaluations related to each formulation (coated tablet and oral solution) defined its position on the map. Similarly, we positioned on the map each formulation according to the sex of the patients (men vs. women). A barycenter, along with the entire 90% confidence ellipsis surrounding it, belonging to the first cluster defining the “Positively accepted” profile could be classified as such. A minimum of 30 evaluations were needed to produce an acceptability score, below this threshold an acceptability tendency could be described. Acceptability could be considered as significantly different if confidence ellipses surrounding two distinct barycenters did not overlap on the map.

Statistical tests were used to assess the significance of the differences observed between the subpopulations of patients in term of age, sex, and disabilities. Pearson’s Chi-squared test was used when there was a minimum expectation of five for 80% of cells without any null expectation [12]. When there were fewer observations, Fisher’s exact test was used.

The R packages “FactoMineR” [13] and “MissMDA” [14] were used to perform multivariate analysis and to handle missing data, respectively (RStudio Version 1.0.136).

### 2.2. Aversiveness Study in Animals

The rodent Brief Access Taste Aversion (BATA) model detects objectively the aversive taste of active pharmaceutical ingredients (API) [15,16,17,18]. This model, usable at an early stage of drug development, seems quite predictive of human taste assessments [19]. It utilizes a lickometer which records the number of licks that rodents (placed in an intermittent water-restriction schedule) take of different test solutions in a standardized protocol [3].

#### 2.2.1. Test Solutions

The original oral solution of memantine (Ebixa^®^) consisted of memantine hydrochloride at a concentration of 10 mg/mL. Sorbitol is present as a sweetener agent, as well as potassium sorbate as a preservative, dissolved in purified water. The oral solution of memantine was assessed in its unadulterated form as it would be administered to patients, and as 3-, 9-, and 27-fold dilutions in deionised water, to examine any potential concentration effect.

The precise quantitative composition of excipients in the original memantine oral solution was unknown. Therefore, solutions of excipients alone—potassium sorbate and sorbitol—were also assessed and are referred to as “placebo” from here on despite not being the strict placebo. Potassium sorbate (0.192% *w*/*v*) and sorbitol (10% *w*/*v*) were thus tested as drug free controls, along with 3- and 9-fold dilutions (in deionised water) of these solutions.

#### 2.2.2. Data Collection

All procedures were carried out in accordance with the Animals (Scientific Procedures) Act 1986 (Project Licence PPL 70/7668), following the experimental method described hereafter.

Ten adult males and 10 adult females Sprague-Dawley rats (Charles-River, Kent, UK) aged 8 weeks old were housed in pairs (same sex) in standard cages in a room that was maintained at 21 ± 2 °C with 55% ± 10% humidity and with a 12:12 h light/dark cycle. All training and testing occurred during the light phase of the cycle. Animals had free access to chow (Harlan, Oxon, UK) and tap water except for training and testing periods where a water-restriction schedule occurred. Each rat was water-deprived for 22 h before each session (training and testing) and was then placed with the lickometer (“Davis MS-160” lickometer from DiLog Instruments—Tallahassee, FL, USA) for a maximum session-length of 40 min. After each session, the rodents received tap water for rehydration. As a safety and welfare measure each animal’s weight was monitored to ensure that it did not drop below 85% of their free feeding weight.

The initial days of the protocol were dedicated to training: on the first training day, the shutter was continually open, presenting a single tube containing deionised water to acclimatize the rodent to the lickometer; while on the second training day, 16 tubes containing deionised water were presented on the moving rack with the shutter opening/closing to acclimatize the rodent to the associated noise.

The training was followed by two testing days during which each rat was presented with various sipper tubes containing either deionised water or one of the test preparations. The samples were both presented at random and arranged at random on the Davis Rig (Table S1). The trial began when the rat took its first lick from the sipper tube and ended 8 s later when the shutter closed. Each concentration was presented four times per session (two bottles per test solution, two presentations per bottle).

#### 2.2.3. Data Analysis

We followed the statistical analysis proposed by Soto et al. [20], as described briefly hereafter. As the data were non-parametric (confirmed by the Shapiro–Wilk test of normality), the Kruskal–Wallis test was performed to check for significant differences in the “lick numbers” between the different test preparations. When significant, this analysis was followed by a post-hoc analysis carried out with the non-parametric multiple test of Gao et al. to determine which test solutions were significantly different.

The percentage of inhibition of licks (/8 s) compared to a reference (i.e., deionised water or placebo formulations) was calculated according to this equation:

$$\%\;inhibition\;of\;licks\;=\;\frac{N_{0}\;licks_{reference}-\;N_{0}\;licks_{concentration\;API}}{N_{0}\;licks_{reference}}\;\times \;100.$$

The R package “nparcomp” was used to compute the simultaneous *p*-values.

Test solutions were classified as fully tolerated, when there is no significant difference compared to the negative control; well tolerated, when licks are significantly suppressed with respect to the control but lick inhibition is lower than 30%; tolerated, when the lick inhibition between 31%–50%; aversive/untolerated, when the lick inhibition is in the range of 51%–75% and; highly aversive/untolerated, when the lick inhibition is greater than 75% [20].

## 3. Results

### 3.1. Acceptability Study in Older Adults

#### 3.1.1. Patient Characteristics

Among the 1517 evaluations included in the multivariate analysis that gave rise to the final acceptability reference framework, 390 (26%) defined the cluster “negatively accepted” (Tables S2 and S3 summarize the characteristics of the patients and the products included in the core study, respectively). The original formulations of memantine were evaluated therein for 85 patients in hospital. The mean age of these patients was 85 (6.6), the minimum age was 70, the maximum was 99, and 63.5% were women.

There were 25 patients treated with the coated tablet (88% receiving 20 mg tablets and 12% receiving 10 mg tablets), while the remaining 60 received the oral solution. The tablets are elliptical varying in length from 10.5 mm (10 mg) to 13.1 mm (20 mg). There were no significant differences between these groups of patients in terms of sex (*p* = 0.85), age group (*p* = 0.33), medicine exposure (*p* = 1), cognitive impairment (*p* = 1), muscular or rheumatologic disorders of the upper limbs (*p* = 1), and swallowing disorder (*p* = 0.72). Polypharmacy—≥10 different pharmaceutical drug products per day—was significantly higher in the patients treated by tablet than in those treated with the oral solution (*p* = 0.03).

#### 3.1.2. Acceptability of the Original Formulations of Memantine

**Figure 1:**
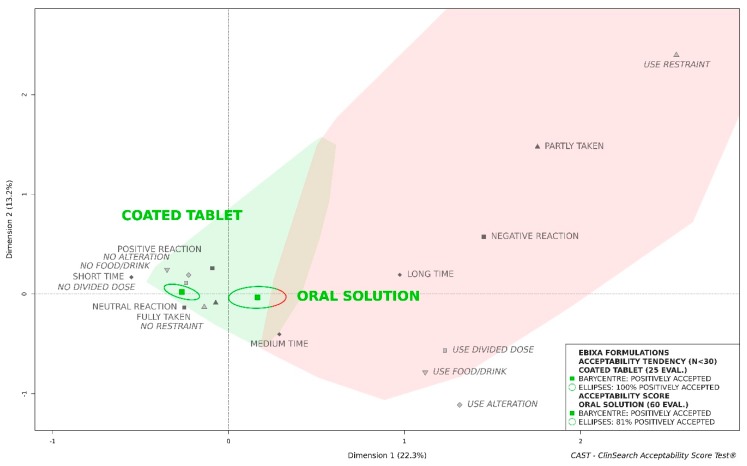
Acceptability of the original formulations of memantine (Ebixa^®^) in the older population.

As the barycenter of all the 25 evaluations of the coated tablet was assigned to the “positively accepted” profile, together with 100% of the surrounding confidence ellipses, this formulation tended to be considered as accepted (see Figure 1). Although the barycenter of all the 60 evaluations of oral solution was assigned to the “positively accepted” profile, a significant part of the confidence ellipses was assigned to the “negatively accepted” profile. These results highlight that in the whole population, regardless of patient characteristics, the oral solution was less well accepted than the coated tablet which presumably masked the aversive taste of the API.

#### 3.1.3. Sex Differences

**Figure 2:**
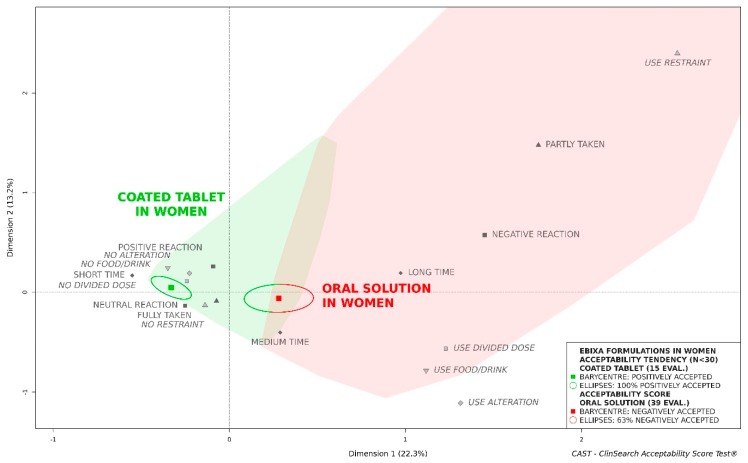
Acceptability of the original formulations of memantine (Ebixa^®^) in older women.

In men, both formulations tended to be similarly accepted, the confidence ellipses of the coated tablet and the oral solution were overlapping. Conversely, the oral solution could not be considered as accepted in women, while the coated tablet tended to be classified as such (Figure 2). There were no significant differences between the groups of patients compared for both sexes, with the exception of more frequent polypharmacy in the women treated by tablet than in those treated with the oral solution (Table 1).

### 3.2. Aversiveness Study in Animals

#### 3.2.1. Aversiveness of Test Solutions

All preparations were assessed by 10 male and 10 female rats, both placebo and the original oral solution of memantine. They were perceived as more aversive than water as indicated by a significant reduction in lick number (Table 2).

The number of licks for each dilution of the original oral solution of memantine were significantly different from each other (Table 2), while no significant differences were observed between the lick numbers measured for the placebo formulation or any of its dilutions (Table 2). Lastly, the lick number of all preparations of the original oral solution of memantine—unadulterated and diluted forms—were significantly lower than those of placebo (Table 2), demonstrating the aversiveness of memantine hydrochloride alone.

All test solutions of the original oral solution of memantine were classified as highly aversive/untolerated (lick inhibition greater than 75%), except the solution diluted 27 times which was classified as aversive/untolerated (lick inhibition in the range of 51%–75%). All the placebos were well tolerated (lick inhibition <30%).

#### 3.2.2. Sex Differences

**Figure 3:**
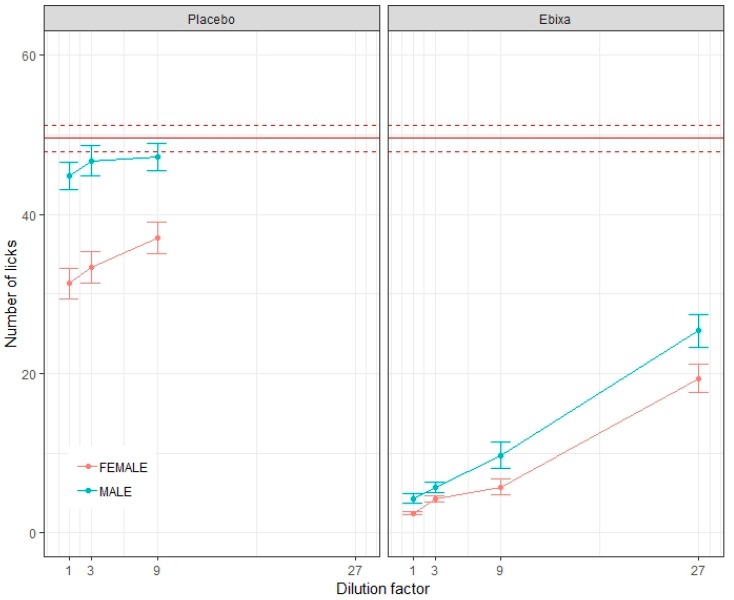
The aversiveness of test solutions for male and female rats. The average lick number for each dilution of placebo and original oral solution of memantine (Ebixa^®^) for male and female rats ± Standard Error of the Mean (SEM), with the average lick number of water shown as a solid red line ± SEM.

No significant differences were observed between male and female rats for water (*p* = 0.12). However, all other formulations yielded significantly different lick numbers among male and female rats (Table 2), with the exception of the 3-fold diluted original oral solution of memantine, where no significant difference was observed (*p* = 0.35); see Figure 3. Lick counts were consistently higher in males for all samples.

## 4. Discussion

These results demonstrated in the older population that palatability influences acceptability. Although oral solutions are often assumed to be better adapted dosage form for this ageing population, coated tablets have proven to be better accepted in this case. Subsequent sub-population analyses of patient characteristics highlighted that sex was an important factor in memantine hydrochloride acceptability. For men, no differences were observed between the oral solution and the tablet (positively accepted), but the results were significantly different among women for whom the oral solution was classified “negatively accepted.”

The characteristics of the women treated with the tablets versus those treated with the oral solution (Table 2) were explored to better understand this result, revealing only that the number of medicines prescribed per day was statistically different between the two groups. As those women taking the oral solution had been prescribed significantly fewer medicines, it would be difficult to consider that receiving more than 10 treatments per day could increase patient acceptability. As patient characteristics did not provide any explanation for this difference in acceptability, product characteristics were explored. Ingestion of the oral solution imposes a direct exposure of the mouth to the dissolved API, whereas the tablet coating provides a physical barrier between the API and the patient’s taste buds [22]. Thus, it is hypothesized that poor acceptability of the oral solution of memantine hydrochloride may have been driven by a palatability issue. While it is well known that palatability optimization of oral solutions of an aversive API is challenging, it was surprising to observe such a gap in acceptability between women and men. For these reasons we further investigated this using the BATA model to compare male and female rats. After having confirmed that the memantine hydrochloride was highly aversive/untolerated for rats in general, we also observed that it was significantly more aversive for female rats than for males.

A recent systematic review focused on acceptability assessment of oral formulations among children and older adults identified that only 17 studies have been included in the analysis of [23]. Even fewer acceptability studies have been conducted in older populations, to the best of our knowledge this is the first study that reveals a sex difference in medicine acceptability.

It would have been interesting to explore further sub-population analyses, but the discontinuation of memantine hydrochloride subsidizing in France in August 2018 for efficacy and safety reasons [24] prohibits further data collection in France. Nonetheless, a substantial number of patients were included in this study, with a low heterogeneity of evaluations within each group. All of the observations included in the analysis were performed in hospitals or nursing homes, while observations of older home dwelling patients are lacking from these analyses. If we consider that the cognitive functions are, per definition, more altered for institutionalized patients, we do not think that the aversiveness for memantine hydrochloride could be lower in ambulatory treated women, but sensitivity to bitterness could be higher for ambulatory men. However, to verify this assumption recruitment would have to include other countries for the aforementioned reason.

The BATA model results are also of interest with regards to the excipients: the “Placebo” solution presented a greater difference of aversiveness between male and female rats than the memantine hydrochloride solution itself. The excipients of the original oral solution of memantine are 10% sorbitol and potassium sorbate in purified water. At least one of these appears to be aversive for the female rats but not for the males. Unfortunately, the experimental design did not allow for each ingredient to be screened separately. However, sex differences in sugar preference have previously been discussed in the literature. One of the studies testing the taste preference for glucose or saccharine solutions demonstrated that female rats were more attracted to sweetness in oral solutions than males [25]. Similar results have been reported in other conditions with food [26]. However, in contrast to bitterness, results related to sweetness and sex seem to be more difficult to extrapolate from this model to human behavior. Effectively, a sensory study in human subjects [27] identified a difference of optimal sweetness level depending on the sex, 20% sucrose for male and 10% for female, but the results of this study also showed that the optimal level of sucrose decreased with age from 20% to 10%. Concerning the second excipient, potassium sorbate is considered to be have a neutral taste [28]. Thus, although it is unlikely to be the aversive factor in our drug free solutions as they were freshly made daily, under some conditions the degradation of this molecule could generate a bitter taste [29] and even a characteristic geranium taste [30]. To better understand their potential role in aversiveness, further relevant investigations should be conducted.

Nevertheless, sex differences appear to be an important factor to consider for dosage form prescription. Unpublished observations on oral liquid solution in older populations and preliminary findings on antibiotics formulations with distinct flavors have highlighted such a sex difference due to excipient compositions.

The human study and animal model demonstrated that the unpleasant taste of the oral solution was a major issue in both women and female rats. The BATA tool generates relevant results in an objective and quantitative manner for the early prediction of aversiveness during drug development. Here, these results were consistent with the observations analyzed by CAST, which offers a suitable scoring tool for the evaluation of medicine acceptability in real-life conditions.

Despite reported observations that Alzheimer disease worsens taste and smell impairments in the older population [31], this study has demonstrated that palatability issues remain an important driver of acceptability in these targeted patients. Palatability should therefore be more broadly monitored during drug development by manufacturers and regulators even when the intended to treat population is an older one.

These findings confirm that acceptability is determined by both users and products characteristics, and underline the need for a better understanding of patients’ needs to promote personalized prescription.

## 5. Conclusions

This study on a particular Alzheimer’s disease treatment highlighted acceptability issues with the original oral solution of memantine (Ebixa^®^) driven by palatability. Indeed, according to CAST the coated tablet, which created a physical barrier between the memantine hydrochloride and taste buds, was well accepted in the older population, while this appeared not to be the case for the oral solution. Furthermore, the BATA model objectively confirmed the aversiveness of this formulation. Exploring sex differences, consistent findings from both human studies and animal models highlighted a higher sensitivity of the females to this unpalatable oral formulation as the proposed cause for suboptimal acceptability. These findings showed that palatability of oral liquid pharmaceutical formulations can remain a key aspect of acceptability in the older population, especially in women. Therefore, formulation scientists and the pharmaceutical industry should consider taste masking as a key quality attribute of all products developed for the older population. Furthermore, healthcare professionals should consider the specific features of their patients to prescribe a medicine with the best adapted characteristics to reach an optimal acceptability.

## Figures and Tables

**Table 1:** Characteristics of the patients treated with the original formulations of memantine (Ebixa^®^).

Patient Characteristics	Women		Men	
	Coated Tablet (*n* = 15)	Oral Solution (*n* = 39)	Coated Tablet (*n* = 10)	Oral Solution (*n* = 21)
**Age (Years)**				
[65, 75[	1 (7) ^1^	4 (10)	0 (0)	3 (14)
[75, 85[	5 (33)	11 (28)	3 (30)	8 (38)
[85, 95[	9 (60)	19 (49)	7 (70)	10 (48)
[95, 104]	0 (0)	5 (13)	0 (0)	0 (0)
Statistical Test	0.642 (F) ^2^		0.557 (F)	
**Disabilities**				
Swallowing disorder	2 (13)	5 (13)	0 (0)	3 (14)
Statistical Test	1 (F)		0.533 (F)	
Muscular or rheumatologic disorders of the upper limbs	2 (13)	5 (13)	1 (10)	3 (14)
Statistical Test	1 (F)		1 (F)	
Cognitive impairment	15 (100)	39 (100)	10 (100)	21 (100)
Statistical Test	1 (F)		1 (F)	
**Medicine Exposure**				
Already taken	15 (100)	39 (100)	10 (100)	21 (100)
**Number of Prescribed Medicines per Day**				
[1–5[	0 (0)	0 (0)	1 (10)	0 (0)
[5–10[	4 (27)	24 (62)	3 (30)	8 (38)
≥10	11 (73)	15 (38)	6 (60)	13 (62)
Statistical Test	0.033 (F)		0.490 (F)	

^1^ number and percentage: *n* (%); ^2^ F: Fisher’s Exact Test *p*-value.

**Table 2:** Non-parametric statistical testing to compare lickometer results between test solutions.

Test Solutions	Water	Ebixa	Ebixa 3FD	Ebixa 9FD	Ebixa 27FD	Placebo	Placebo 3FD	Placebo 9FD
**Water**	NA	<0.001	<0.001	<0.001	<0.001	<0.001	<0.001	<0.001
**Ebixa**	<0.001	NA	0.013	<0.001	<0.001	<0.001	<0.001	<0.001
**Ebixa 3FD**	<0.001	0.025	NA	0.048	<0.001	<0.001	<0.001	<0.001
**Ebixa 9FD**	<0.001	<0.001	0.048	NA	<0.001	<0.001	<0.001	<0.001
**Ebixa 27FD**	<0.001	<0.001	<0.001	<0.001	NA	<0.001	<0.001	<0.001
**Placebo**	<0.001	<0.001	<0.001	<0.001	<0.001	NA	0.44	0.14
**Placebo 3FD**	<0.001	<0.001	<0.001	<0.001	<0.001	0.44	NA	0.44
**Placebo 9FD**	<0.001	<0.001	<0.001	<0.001	<0.001	0.14	0.33	NA
**Males vs. Females**	0.12	<0.001	0.35	0.008	0.032	<0.001	<0.001	<0.001

Results of post-hoc analysis carried out with Gao et al.’s non-parametric multiple test [21] to compare lickometer results between Ebixa^®^ (pure, at 3-, 9-, and 27-fold dilutions) and drug-free solutions (deionised water, and “placebo” and its dilutions containing excipients alone), as well as any observed differences between the males and females for each solution tested (*p* < 0.05 may be considered as significative).

## Supplementary Materials

The following are available online at https://www.mdpi.com/1999-4923/11/8/368/s1, Table S1: Randomization schedule for testing days 1 and 2, Table S2: Demographic characteristics of the patients, Table S3: Characteristics of the medicines.
