# Supplementary material for: Sex Differences in Medicine Acceptability: A New Factor to Be Considered in Medicine Formulation
Source: Pharmaceutics. 2019 Aug 1;11(8):368. doi: 10.3390/pharmaceutics11080368 (PMC6723034; doi:10.3390/pharmaceutics11080368)
Supplement: Supplementary file 1 [file pharmaceutics-11-00368-s001.pdf]

pharmaceutics

Supplementary materials: Sex Differences in Medicine Acceptability: A New Factor to Be Considered in Medicine Formulation

Fabrice Ruiz, Alexander Keeley, Patrick Léglise, Catherine Tuleu, Célia Lachuer, Jean-Paul Rwabihama, Nathalie Bachalat, Imad Boulaich, Fattima Abdallah, Maité Rabus, Annie-Claude Ribemont, Hugues Michelon, Amélie Dufaÿ Wojcicki, Mine Orlu, Thibault Vallet and Vincent Boudy

Table S1. Randomisation schedule for testing days 1 and 2.

Tube Number	Testing Day 1	Testing Day 2
1	Ebixa® 9FD	Ebixa® 27FD
2	Ebixa® 9FD	Ebixa® 27FD
3	Water Control	Water Control
4	Placebo	Ebixa® 3FD
5	Ebixa® 27FD	Ebixa®
6	Placebo	Ebixa® 9FD
7	Ebixa®	Ebixa® 3FD
8	Water rinse	Water rinse
9	Ebixa® 3FD	Placebo
10	Placebo 3FD	Placebo 9FD
11	Placebo 9FD	Placebo 3FD
12	Ebixa® 3FD	Placebo
13	Placebo 3FD	Ebixa® 9FD
14	Placebo 9FD	Placebo 9FD
15	Ebixa® 27FD	Ebixa®
16	Ebixa®	Placebo 3FD

Table S2. Demographic Characteristics of the Patients.

Patients (n = 1517)			
Characteristics		n	(%)
**Sex**	Women	1043	(69)
	Men	459	(31)
	md [a]*: 15*		
**Age (years)**	[65, 75[	88	(6)
	[75, 85[	463	(31)
	[85, 95[	777	(51)
	[95, 104]	183	(12)
	md: 6		
**Place**	Hospital	1274	(84)
	Nursing home	243	(16)
**Disabilities**	Swallowing disorder	279	(19)
	Muscular or rheumatologic disorders of the upper limbs	301	(20)
	Cognitive impairment	882	(59)
**Number of prescribed medicines per day**	[1–5[	99	(7)
	[5–10[	661	(44)
	≥10	738	(49)
	md: 19		

[a] *md: missing data.*

Table S3. Characteristics of the Medicines.

Medicines (n = 355)			
**Characteristics**		***n***	**(%)**
**Formulations**	Coated tablet	58	(16)
	Divisible tablet	48	(14)
	Divisible coated tablet	41	(12)
	Capsule	44	(12)
	Tablet	22	(6)
	Oral solution	18	(5)
	Other (2% < n < 5%): orally disintegrating tablet, powder for oral solution, prolonged release tablet, prolonged release capsule, ocular solution		
	Other ($n \leq 2\%$): oral suspension, coated prolonged release tablet, dispersible tablet, transdermal patch, divisible effervescent tablet, drops for oral solution, powder for oral suspension, solution for injection, effervescent tablet, gastro resistant capsule, solution for injection in pre-filled pen, divisible coated prolonged release tablet, gastro resistant tablet, powder for inhalation, suspension for inhalation, syrup, coated gastro resistant tablet, dispersible coated tablet, dispersible or chewable tablet, divisible tablet for oral suspension, emplatre, injection and oral solution, ocular gel, oral gel, powder for inhalation in capsule, powder for oral and rectal solution, powder for solution for injection, prolonged release granules, soft capsule, solution for inhalation, suckable tablet, suspension for injection in pre-filled pen, sustained release microgranules in capsule		
**Anatomic therapeutic subgroups (ATC level 2)**	Analgesics	44	(12)
	Psycholeptics	41	(12)
	Psychoanaleptics	40	(11)
	Antiepileptics	30	(8)
	Drugs used in diabetes	20	(6)
	Other (2% < n < 5%): agents acting on the renin-angiotensin system, antithrombotic agents, beta blocking agents, anti-parkinson drugs, calcium channel blockers, drugs for constipation, antibacterials for systemic use, ophthalmologicals		
	Other ($n \leq 2\%$): antianemic preparations, drugs for acid related disorders, lipid modifying agents, mineral supplements, drugs for obstructive airway diseases, corticosteroids for systemic use, diuretics, other nervous system drugs, urologicals, all other therapeutic products, antihypertensives, antivirals for systemic use, cardiac therapy, vitamins, anesthetics, antidiarrheals intestinal anti inflammatory anti infective agents, antigout preparations, antihemorrhagics, anti inflammatory and antirheumatic products, antimycobacterials, antimycotics for systemic use, antiprotozoals, drugs for functional gastrointestinal disorders, endocrine therapy, muscle relaxants, vasoprotectives, thyroid therapy		
